# Comparison of the activity and pluripotency maintaining potential of human leukemia inhibitory factor (LIF) produced in *E.coli* and CHO cells

**DOI:** 10.1186/1753-6561-5-S8-P109

**Published:** 2011-11-22

**Authors:** Claas Haake, Sophia Bonk, Jana Parsiegla, Magda Tomala, Komal Loya, Malte Sgodda, Tobias Cantz, Axel Schambach, Cornelia Kasper, Thomas Scheper

**Affiliations:** 1 Institute for Technical Chemistry, Leibniz University of Hanover, 30167 Hannover, Germany; 2 Junior Research Group Stem Cell Biology, Cluster of Excellence REBIRTH; MPI Münster and Hanover Medical School, Hannover Germany; 3 Department of Experimental Hematology, Hanover Medical School, 30625 Hannover, Germany

## Introduction

Leukemia inhibitory factor (LIF) is a polyfunctional cytokine with numerous regulatory effects *in vivo* and *in vitro*. In murine stem cell cultures it is the essential media supplement for the maintenance of pluripotency of embryonic and induced pluripotent stem cells. To explore if the glycosylation and/or other post-translational modifications are affecting this activity, we produced and isolated LIF from either eucaryotic cells (Chinese hamster ovary (CHO) cells) or procaryotes (*E.coli*) and compared their biological activities in this study.

## Results

### Production in E.coli

To increase the solubility the LIF is expressed together with thioredoxin as a fusion protein. The genes for the fusion protein are separated on the vector (pET32b) by a TEV (Tobacco Etch Virus) protease cleavage site, in addition thioredoxin is expressed with a his-tag. The resulting construct was transformed into *E.coli* BL21(DE3), which were afterwards cultivated at 23°C. This relatively low temperature leads to increased solubility and yield of the target protein. The protein was afterwards purified from the fermentation broth by metal chelate affinity chromatography through the utilization of Zn^2+^ ions immobilized on IDA membrane adsorbers. To cleave the thioredoxin from the fusion protein the obtained protein fractions were directly treated with recombinant TEV protease. The released hLIF was purified from the protein mixture using ion exchange chromatography [1].

### Production in CHO cells

The utilized CHO^SFS^ cell line (CCS, Hamburg, Germany) was transducted using a lentivirus with a pRRL vector, which coded for his-tagged humane LIF. Verification of the expression, as well as of the extracellular localization was carried out by intracellular flow cytometric staining and by western blot. The cultivation in serum free medium (ProCHO5, Lonza, Basel, Switzerland) was initially performed in spinner flasks. Afterwards the scale-up to 1.5 L bioreactor scale was accomplished. The cultivation was carried out at 37°C, 200 rpm, and pH 7.2. The first step of the purification of the concentrated supernatant was performed by metal chelate affinity chromatography, where Ni^2+^ ions were immobilized. Afterwards the pooled protein fractions were purified by a polishing step by heparin affinity chromatography.

### Effects on murine iPS suspension cells

*Cell growth*: The cells were cultivated as cell spheres in suspension in DMEM medium supplied with 10 ng LIF per ml. As positive control ESGRO LIF, which is produced in *E.coli*, was used. For each of the three LIFs used as well as for the negative control two cultures of 2 ml each were harvested using trypsin every day and the cell count was determined manually using trypan blue. For the long-term cultivation the cells were passaged twice a week. The obtained values for maximal cell density and the viabilities show the activity of the produced LIFs when compared with the positive and negative control but no discrepancy in their activities.

*Pluripotency maintaining potential*: Via flow cytometry the expression of the pluripotency markers SSEA-1 and Oct4 was determined using an anti-SSEA-1-PE antibody and measuring the expression of GFP, which stands under the control of an Oct4 promoter respectively. For the negative control an isotype control antibody was used for SSEA-1. For GFP, the results achieved were compared to the GFP expression of the cells at the start of the cultivation. The results show a slight decrease in SSEA-1 (FL-2) but not GFP (FL-1) expression over the duration of the cultivation, but no differences regarding the activities in accordance to the source of the LIFs.

### Effects on adherent growing murine ESC

Murine ESC, carrying an Oct4-GFP reporter construct (OG2-ESC) [2] were seeded on C3H irridatred mouse embryonic fibroblast cells in 6-well-plates with LIF concentrations of 40, 10, and 2.5 ng/ml. As positive control an *E.coli*-derived LIF was used, which has been produced at the MPI Münster and has previously been tested. The media were changed every 2 days and the cells were passaged twice a week for 5 passages. After 24 days the cells were harvested and used for qRT-PCR. Within the qRT-PCR β-Actin was used as the housekeeping gene. The expression of the pluripotency markers Oct4 and Nanog as well as marker proteins for the three germ layers (Trp63 for ectodermal, AFP for endodermal and Brachyury for mesodermal differentiation) were quantified. The results show that the mesodermal marker is downregulated, which indicates spontaneous differentiation of the negative control into the mesodermal germ layer. Between the used LIFs no significant differences could be determined.

### Effects on murine ES suspension cells

**Figure 1:**
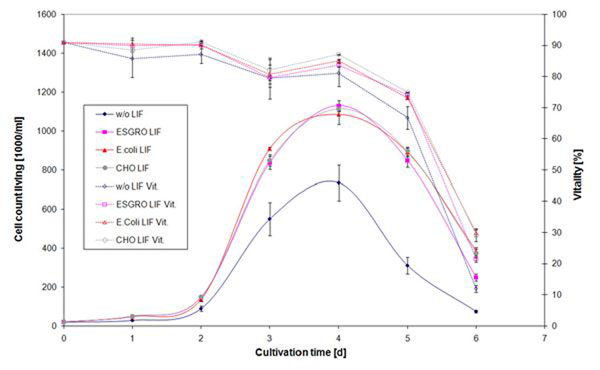
Living cell count and viability against the cultivation time for the cultivation of the ES cells with 10 ng LIF/ml.

*Cell growth*: The procedure was in accordance to the approach with the iPS cells and the three LIFs were also used in concentrations of 100 and 1 pg each. Supplementary the amount of apoptosis was measured after four days of culture using the Annexin-V Kit (Invitrogen, Carlsbad, CA, USA) for flow cytometry. The results clearly show activity for the concentrations of 10 ng (figure 1) and 100 pg but no considerably difference from the negative control for the concentration of 1 pg and also no differences for the LIFs regarding their sources were found.

*Pluripotency maintaining potential*: The marker protein SSEA-1 was measured flow cytometrically once a week for the culture containing 10 ng/ml LIF as well as once after 14 days for the one containing 1 pg. The SSEA-1 expression remains at a level of above 97% when 10 ng LIF/ml were used, but is broadly decreased for the negative control. For the concentration of 1 pg LIF/ml SSEA-1 expression levels of about 90% after 14 days were achievable although this amount of LIF showed no differences from the negative control in regards of cell viability. From these results we can state that with regard to the pluripotency maintaining potential the different LIF did not show detectable differences.

## Conclusion

The production and isolation of LIF from *E.coli* as well as from CHO cells was successfully established. The bioactivity of both proteins was demonstrated in various experiment using different cells and methods of detection. We conclude from our results that LIF from mammalian cells and LIF from prokaroytes are equally effective.
